# Implementation of Parameter Observer for Capacitors

**DOI:** 10.3390/s23020948

**Published:** 2023-01-13

**Authors:** Corneliu Bărbulescu, Dadiana-Valeria Căiman, Sorin Nanu, Toma-Leonida Dragomir

**Affiliations:** Department of Automation and Applied Informatics, Politehnica University Timișoara, Bulevardul Vasile Pârvan, nr. 2, 300223 Timișoara, Romania

**Keywords:** parameter observer, electrolytic capacitor, equivalent capacitance, equivalent series resistance, discrete-time systems, implementation on microcontroller

## Abstract

This paper describes the implementation of a parameter observer (*PO*) intended to estimate the capacitance and equivalent serial resistance of a capacitor (ESR). The implemented observer consists of a dynamic second-order discrete-time system. The input signal of the observer is the voltage at the terminals of the capacitor measured during its discharge across a variable resistance in two steps. The implemented observer can be used in quasi-online or offline mode. The theoretical and experimental supporting materials provide a comprehensive picture of the implementation and conditions of use of the *PO*. The experimental verification was carried out with a microcontroller with Cortex^®^-M7 core architecture. The sampling time of the *PO* was 20 μs, and the estimation of the parameters was obtained before the end of the discharge of the capacitor. In the cases described in the paper, this means approximately 25 ms. Due to the *PO*’s capabilities (estimation speed, reduced computational complexity and precision)—proved by the experiments carried out on three electrolytic capacitors of 100 μF, 220 μF and 440 μF—the implementation is of interest for several applications, primarily in the field of power electronic applications.

## 1. Introduction

Electric capacitors are passive electrical devices that are indispensable for electrical circuits in electronic applications, hereinafter referred to as processes. In designing the circuits, depending on the operating regimes to which the capacitors are subjected in the processes, we use several topologies of electrical models, from the simple capacity, or capacity in series with a resistance, to models with more capacities, resistances and inductances. In this context, the parameters of the models are called equivalent parameters of a capacitor.

In many processes, capacitors play key roles in filtering and storing energy, being indispensable elements. As an example, we mention the aluminum electrolytic capacitors used in power electronic converters and in the circuits of photovoltaic panels (solar PV). In general, we must refer to their applications in power electronics [1,2,3]. The use and ageing of the capacitor changes the values of its parameters over time and thereby renders it vulnerable in fulfilling its role. The modification alters the performance of processes, leading to their failure. When referring to the R-C series electrical model of a capacitor, the change manifests as a decrease in the value of the equivalent capacitance and an increase in the ESR. Consequently, these two parameters are considered indicators of the state of the capacitor, and monitoring their values is vital [4]. An ample specialized literature deals with this topic. As the case may be, the approaches are restricted to monitoring or are integrated into applications based on monitoring, e.g., identification, capacitor health monitoring, fault diagnosis, operational safety assurance, parameter variation compensation, etc. [1,5,6,7,8,9,10].

Various principles are used for monitoring the equivalent parameters of capacitors. One of these is based on the use of observers [11]. Usually, observers are used to dynamically estimate either endogenous or exogenous state variables of processes, combinations thereof, or signals that are functions of state variables. Observers are used for a large range of applications, including in the field of sensors. For instance, the authors of [12] proposed a nonlinear observer for the estimation of the current ripple in a ferrite-core inductor, and the work in [13] introduced a dual-observer to estimate the filter output current and voltage for a sensorless field-oriented control drive. Different from the observers mentioned, the authors of [11] recently proposed a parameter observer for estimating the equivalent values *C_e_* and *R_se_* of an R-C serial model of a capacitor. This does not estimate the state variables, but rather the time constants of several first-order linear circuits that appear during the two-stage discharge of the capacitor. From a dynamic point of view, the discharge corresponds to the free regime of a first-order linear system, the signal having the dynamic characteristics of the system’s impulse response. The parameter observer is a second-order dynamic system in discrete time that has a single input signal, namely, the one for which it calculates the equivalent time constant. The use of a single input signal makes it more reliable and less vulnerable to disturbances.

Often, we investigate the behavior of capacitors using the frequency characteristics of impedance, capacitance and ESR, i.e., *Z*(*f*), *C*(*f*) and *R_se_*(*f*), respectively [14,15,16]. The fact that *C*(*f*) and *R_se_*(*f*) are not constant shows that the parameters of the capacitor change during dynamic operating regimes, whereby the voltage across the capacitor terminals varies in time. In this context, the “equivalent” attribute takes on an additional connotation, referring not only to the model associated with the capacitor and to its operating regime.

From the perspective of the evaluation of capacitors in relation to the processes in which they are involved, evaluation methods of capacitor parameters are considered to be of three types: online, off-line and quasi-online [17]. In principle, online methods are those that do not involve interventions to separate the capacitor from the process, off-line methods assume the detachment of the capacitor from the process, and quasi-online methods require a short-term interruption of the process without requiring interventions in the process. This last type includes, for example, all processes that do not have a continuous operation.

This paper presents a real-time implementation of the *PO* proposed in [11]. The implementation concerns the quasi-online type of application. The main contribution of this paper is to design the *PO* implementation structure and to illustrate its capabilities in real cases. The novelties introduced in this paper compared to [11] are:

- The implementation of a *PO* on a microcontroller and its validation on electrolytic capacitors.
- The real-time estimation of the values *C_e_* and *R_se_* of the capacitor during the discharge process, which is about 20 ms.
- An improvement of the estimation method for the time-equivalent constants used to calculate *C_e_* and *R_se_*.

The remainder of the article is organized as follows. In Section 2, the underpinning theory of the *PO* is presented synthetically, and the hardware and software aspects related to the implementation on a microcontroller are analyzed in detail. Section 3 presents the implementation of the *PO* in the case of three different capacitors and summarizes the experimental results. Section 4 includes discussions on the experimental results, emphasizing some aspects of *PO* implementation in applications. Section 5 concludes the entire paper by highlighting the *PO*’s expediency.

## 2. Materials and Methods

### 2.1. Theoretical Support

#### 2.1.1. Determining the Values of Equivalent Parameters of a Capacitor Using the Two-Step Discharge Method and a Discrete-Time Parameter Observer

In this section, we summarize under points 1 and 2 the results from [11], which represent the theoretical support of this paper.

1. The time-varying parameter *T*(*t*) of the non-autonomous, first-order, unforced dynamical system (1) with properties (2) can be determined using the discrete-time parameter observer (3).

(1) $$T\left(t\right)\cdot \dot{y}\left(t\right)+y\left(t\right)=0,y\left(0\right)>0.$$

(2) $$y\left(t\right)>0,\dot{y}\left(t\right)<0.$$

(3) $$PO-T:\{\begin{array}{cc} \mathrm{input}\;\mathrm{adapter}(IA): & z[k]=\ln(y[k]),\\ \mathrm{modified}\;\mathrm{model}(M): & \hat{z}[k]=\hat{z}[k-1]+h\cdot \hat{c}[k],\;\hat{z}[0]={\hat{z}}_{o},\\ \mathrm{tracking}\;\mathrm{error}: & \varepsilon [k]=z[k]-\hat{z}[k],\\ \mathrm{tracking}\;\mathrm{controller}(TC): & \hat{c}[k]=K_{p}(\varepsilon [k]-\varepsilon [k-1])+hK_{i}\varepsilon [k-1]+\hat{c}[k-1],\;\hat{c}[0]={\hat{c}}_{o},\\ \mathrm{output}\;\mathrm{controller}(OA): & \hat{T}[k]=-\frac{1}{\hat{c}[k]}. \end{array}$$

**Figure 1:**
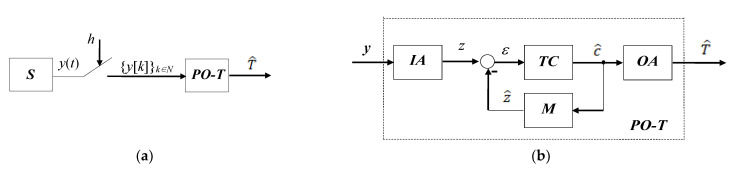
Block schemes: (**a**) the connection between the observed system and the *PO*; (**b**) the structure of the *PO*.

Figure 1 contains the block diagrams associated with these equations. The *S* and *PO-T* blocks in Figure 1a correspond to the Equations (1) and (3), respectively, with $\hat{T}$ being the estimate of T. In (3), $y\left[k\right]$ represents the sample of $y\left(t\right)$ at the moment t=k·h, h being the constant sampling period of the system, and k∈N. The block diagram in Figure 1b shows the structure corresponding to Equation (3) of *PO-T*.

Since $T\left(t\right)$ is unknown, and the value $y\left(0\right)$ is not fixed, the values ${\hat{z}}_{o}$ and ${\hat{c}}_{o}$ are initialized arbitrarily. Consequently, ${\left\{\hat{T}\left[k\right]\right\}}_{k\in N}$ calculated by *PO* will always contain a transient interval to be omitted. For this reason, only the discrete time signal ${\left\{\hat{T}\left[k\right]\right\}}_{k\geq k_{o},k\in N}$ is considered for later use. The value of $k_{0}$ shall be chosen in such a way that from this moment on, the transient interval is practically finished.

2. The main application considered in [11] is to determine the equivalent capacitance $C_{e}$ of a capacitor during a discharging process over a resistor, when the variation in the voltage at the capacitor terminals is assimilated to $y\left(t\right)$ in Equation (1).

As shown in [14], the procedure takes into account the capacitance and ESR variations during the discharging processes. The value of $C_{e}$ is calculated from two equivalent values ${\hat{T}}_{e1}$ and ${\hat{T}}_{e2}$ of $T\left(t\right)$ corresponding to a two-stage capacitor discharge produced by a stepwise modification of the resistance of the discharge resistor. The “equivalent” attribute refers to the fact that, replacing $T\left(t\right)$ from Equation (1) with the values ${\hat{T}}_{e1}$ and ${\hat{T}}_{e2}$, the solutions of Equation (1) on the calculation intervals approximate very well the variations in $y\left(t\right)$ over the corresponding time intervals. If the discharge resistance has the values $R_{ext1}$ and $R_{ext2}$, respectively, during the two stages, then the capacitor’s equivalent capacitance ${\hat{C}}_{e}$ and the equivalent resistance ${\hat{R}}_{se}$ (i.e., ESR) are obtained using Equation (4).

(4) $${\hat{C}}_{e}=\frac{{\hat{T}}_{e1}-{\hat{T}}_{e2}}{R_{ext1}-R_{ext2}},{\hat{R}}_{se}=\frac{{\hat{T}}_{e1}\cdot R_{ext2}-{\hat{T}}_{e2}\cdot R_{ext1}}{{\hat{T}}_{e2}-{\hat{T}}_{e1}}.$$

**Figure 2:**
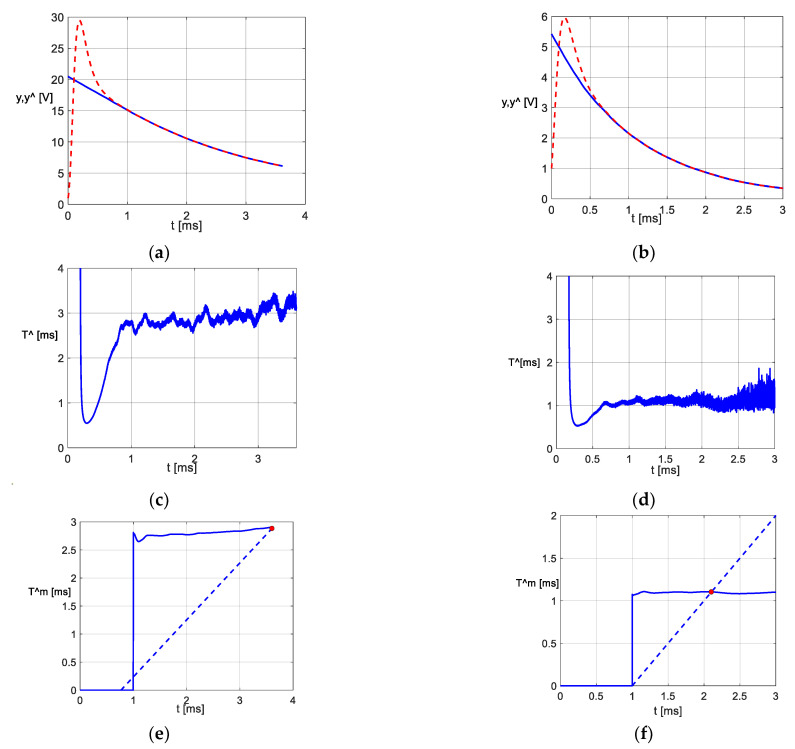
Obtaining the equivalent values ${\hat{T}}_{e1}$ and ${\hat{T}}_{e2}$ using *PO−T*. (**a**) The first stage of discharge; (**b**) the second stage of discharge; (**c**) the variations in ${\left\{\hat{T}\left[k\right]\right\}}_{k\in N}$ for the first discharging stage; (**d**) the variations in ${\left\{\hat{T}\left[k\right]\right\}}_{k\in N}$ for the second discharging stage; (**e**) the variations in ${\left\{{\hat{T}}_{m}\left[k\right]\right\}}_{k\geq k_{o}}$ for the first discharging stage; (**f**) the variations in ${\left\{{\hat{T}}_{m}\left[k\right]\right\}}_{k\geq k_{o}}$ for the second discharging stage.

Figure 2a,b exemplify, according to [11], the two discharge stages of an electrolytic capacitor of 100 μF over a variable resistor, with $R_{ext1}=29.89$ Ω and $R_{ext2}=10.35$ Ω. The voltage $y\left(t\right)$ measured at the capacitor terminals is represented in blue, and its estimated $\hat{y}\left[k\right]=\exp\left(\hat{z}\left[k\right]\right)$ is represented in red. Figure 2c,d show the observer’s response ${\left\{\hat{T}\left[k\right]\right\}}_{k\in N}$ to these input signals for *K_p_* = 2 × 10^4^ s^−1^, *K_i_* = 10^8^ s^−2^ and h=0.5 μs. We remark that the signal $\left\{\hat{y}\left[k\right]\right\}$ does not instantly follow the measured value $y\left(t\right)$. The mentioned transient process occurs. Its extended span, $t_{0}$, can be influenced by the values of *K_p_* and *K_i_*. The values of $t_{0}$ and $k_{0}$ are related by the formula $k_{0}=[\frac{t_{0}}{h}]$. For the above example, we considered $t_{0}=1\;\mathrm{ms}$. At the same time, we observe that ${\left\{\hat{T}\left[k\right]\right\}}_{k\geq k_{o},k\in N}$ displays oscillations. They are caused by measurement and processing noises. To attenuate these oscillations, we complete the output adapter with the averaging operation (5).

(5) $${\hat{T}}_{m}\left[k\right]=\frac{1}{k-k_{0}}\cdot \sum_{k_{0}+1}^{k}\hat{T}\left[k\right],k=k_{0}+1,k_{0}+2,\ldots \;.$$

Thus, we obtain the variations in Figure 2e,f.

Under the conditions of the experiments performed in [11], we determined the values of ${\hat{T}}_{e1}$ and ${\hat{T}}_{e2}$ with the “bisector method”. This method states that ${\hat{T}}_{e1}$ and ${\hat{T}}_{e2}$ take the values of ${\hat{T}}_{m}$ from the points marked in red in Figure 2e,f. The application of the bisector method is detailed in Appendix A.

#### 2.1.2. The Influence of the Estimation Accuracy of ${\hat{T}}_{e1}$ and ${\hat{T}}_{e2}$ on the Calculated Values of the Capacitor Parameters

Appendix B presents a study concerning the impact of estimation errors of the values of ${\hat{T}}_{e1}$ and ${\hat{T}}_{e2}$ on the equivalent values $C_{e}$ and $R_{se}$ calculated in accordance with Equation (4). The conclusions that emerge based on this study are the following:

- To measure the equivalent parameters of capacitors by the method of discharging capacitors in two stages over external resistors, as presented in [11,18], the values of ${\hat{T}}_{e1}$ and ${\hat{T}}_{e2}$ must be estimated with the best possible accuracy.
- As the accuracy of calculating the values of ${\hat{C}}_{e}$ is much higher than that of calculating the values of ${\hat{R}}_{se}$, the method is suitable for both *measuring* the value of $C_{e}$ and for *monitoring* the values of $C_{e}$.
- Under the assumption that the deviations $\Delta {\hat{T}}_{e1\%}$ and $\Delta {\hat{T}}_{e2\%}$ are kept within constant but restricted limits, the method can also be used for monitoring the variations in $R_{se}$, the monotony of its variations being maintained over time.

### 2.2. Implementation of the Parameter Observer

#### 2.2.1. *PO−T* Implementation Flowchart

**Figure 3:**
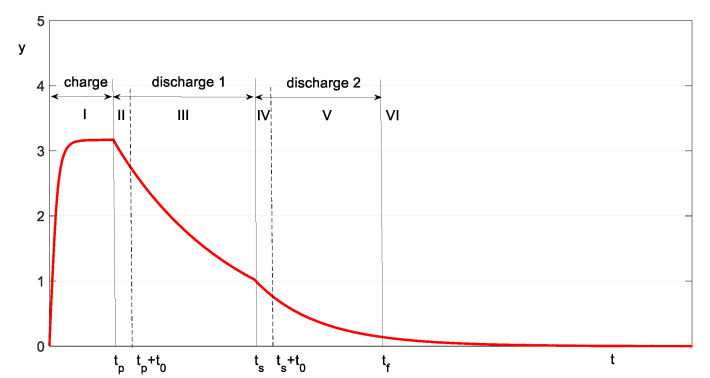
Time diagram of the capacitor charging–discharging process *y*(*t*).

Figure 3 shows the time diagram of the online calculation method of the $C_{e}$ and $R_{se}$ parameters of a capacitor, corresponding to the method presented in Section 2.1.1. The charging of the capacitor occurs in time interval I, and the first discharge occurs at time intervals II and III. The second discharge stage corresponds to intervals IV and V. The intervals II and IV correspond to the transient regimes of *PO-T* and are not used for calculating the parameters. The intervals III and V are used to calculate $T_{e1}$ and $T_{e2}$, respectively. At the end of interval V, we proceed with the calculation of the capacitor’s parameters.

**Figure 4:**
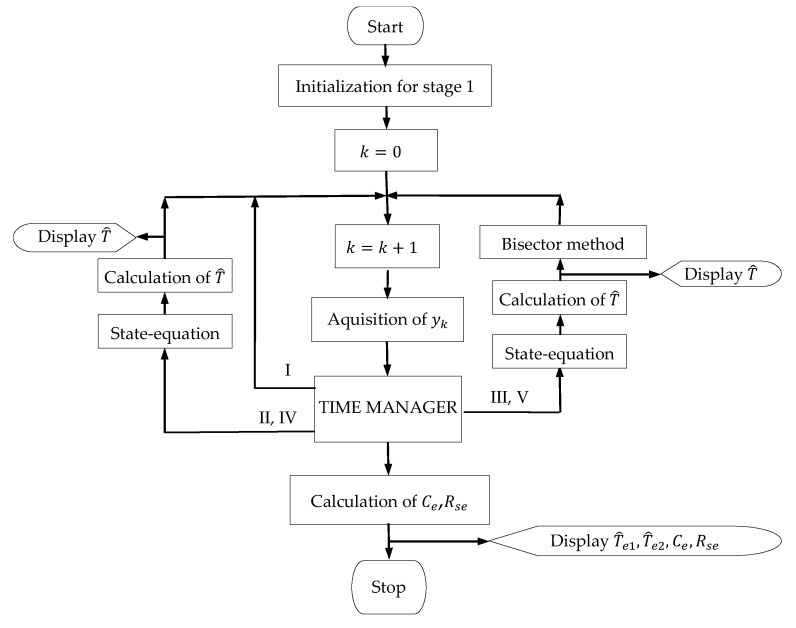
Flowchart of $C_{e}$ and $R_{se}$ parameter calculus method.

The application of the time diagram is carried out according to the simplified flowchart in Figure 4. The TIME MANAGER block manages the discrete time k corresponding to the continuous time t with the sampling period h. As a result, in the time diagram, the values $0,t_{p},t_{p}+t_{0},\cdots ,t_{f}$ are multiples of k according to the equation t=k·h. In each sampling period, the input signal y is acquired (“Acquisition of $y_{k}$” block). In the intervals II–IV, $\hat{T}$ is calculated using Equation (3). The values ${\hat{T}}_{e1}$ and ${\hat{T}}_{e2}$ are calculated in the intervals III and V, respectively, with Equation (5) and the method detailed in Appendix A.

**Figure 5:**
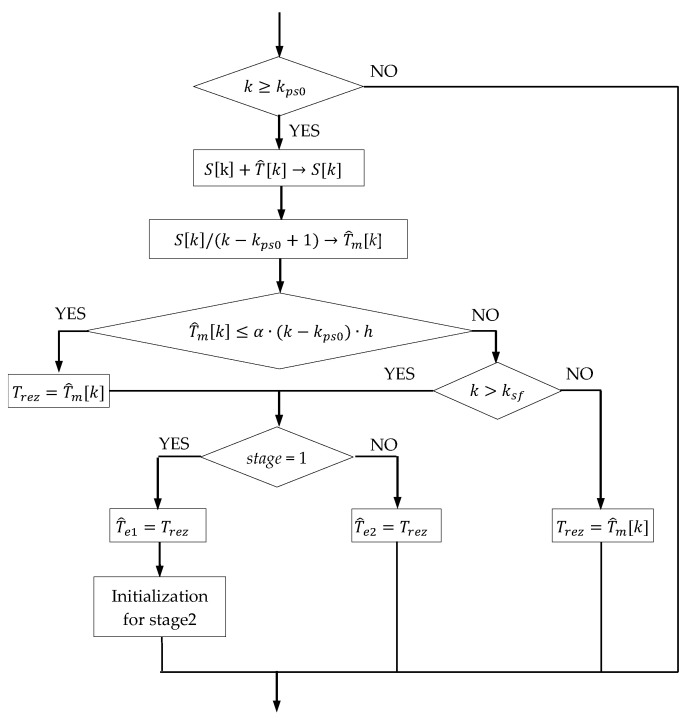
Flowchart for the calculation of ${\hat{T}}_{e1}$ and ${\hat{T}}_{e2}$.

Figure 5 shows the simplified flowchart for the calculation of ${\hat{T}}_{e1}$ and ${\hat{T}}_{e2}$. The parameter *stage* is initialized with the value 1, which is maintained during the first download stage. Later, when new variables are initialized in the second stage, it takes the value 2. The parameter $k_{ps0}$ takes the value $k_{p0}$ corresponding to the moment $t_{p}+t_{0}$ when *stage* = 1 and the value $k_{s0}$ corresponding to the moment $t_{s}+t_{0}$ when *stage* = 2, respectively. Likewise, the parameter $k_{sf}$ takes the value $k_{s0}-k_{o}$ corresponding to the moment $t_{s}$ when *stage* = 1 and the value $k_{f}$ corresponding to the moment $t_{f}$ when *stage* = 2, respectively. $T_{rez}$ represents an internal variable used to memorize the $T_{e}$’s values. The variable $S\left[k\right],$ representing the sum in Equation (5), is initialized as 0 in both stages.

#### 2.2.2. Hardware Support

**Figure 6:**
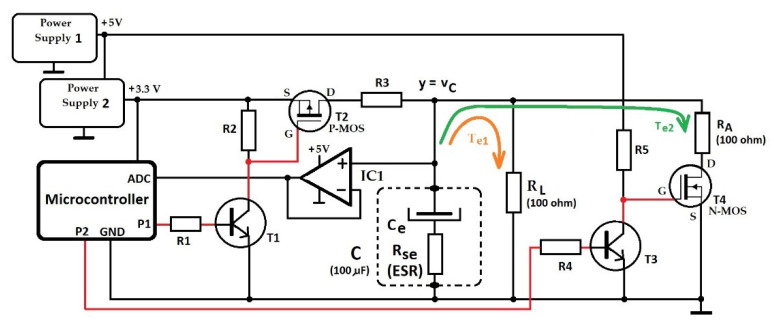
Schematic of the experimental setup.

**Figure 7:**
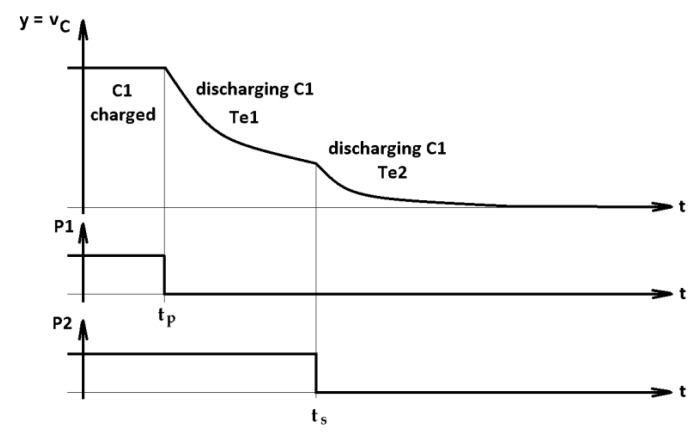
The input and the output signals of the microcontroller in Figure 6.

The schematic used to measure the equivalent capacitance and ESR of a capacitor is represented in Figure 6. It is based on an i.MX RT1062 microcontroller with a Cortex^®^-M7 of NXP core architecture [19], which assures, through the outputs P1 and P2, the control of the capacitor’s discharge process. The control signals and the output signal are shown in Figure 7. The numerical values in this subsection refer to capacitor 1C from Section 3.

The input signal $y=v_{C}$ is acquired and converted into digital format. Then, the microcontroller, according to the *PO-T* algorithm, processes it. To reduce the influence of the input impedance of the microcontroller’s sampling circuit over the discharge process, we inserted the operational amplifier IC1 as a voltage repeater with high input impedance and low output impedance. The IC1 is powered from +5 V supply, assuring the expected linearity in the computed range $\left(0.6÷3.3\;V\right)$. The microcontroller includes a single- and a double-precision floating-point calculation architecture corresponding to the VFPv5 generation, ensuring a fast execution time for the most complex mathematical operations corresponding to *PO-T*. For example, for calculating a logarithm of a real simple precision number (float), the execution time is about 1 μs.

Before the beginning of the discharge, the signal P1 ensures the saturation of the bipolar transistor T1 that keeps the P-MOS transistor T2 open, and thus, the capacitor C is charged through the resistor R3 until time $t_{p}$. Simultaneously, the P2 signal keeps the transistor T3 saturated and the N-MOS transistor T4 consequently blocked, disconnecting the resistor $R_{A}$ from parallel connection with capacitor C. In this way, the charging of the capacitor, up to the moment $t_{p}$, is achieved with the electric current provided by the Power Supply 2 voltage source (+3.3 V) passing through T2 and R3.

At the moment $t_{p}$, the microcontroller blocks the transistor T1 through the signal P1, and the transistor T2 stops the charging of the capacitor C followed by its discharging through the load resistor $R_{L}$ resulting in the first discharge stage. Within this, the discharging process is characterized by the time constant $T_{e1}$ determined by the capacitance $C_{e}$, serial resistance $R_{se}$ of the capacitor C and the resistance $R_{L}=100\;\Omega$.

At the moment $t_{s}$, the microcontroller blocks the transistor T3 through the signal P2 and consequently opens the transistor T4 that connects the additional resistor $R_{A}=100\;\Omega$ in parallel with the load resistor $R_{L}$. Thus, starting with moment $t_{s}$, the process continues with the second discharge stage with a lower time constant $T_{e2}$ determined by a lower equivalent resistance ($R_{L}$ in parallel with $R_{A}$). The Power Supply 1 $\left(+5\;V\right)$ is used to ensure a higher voltage level on the gate of the transistor T4, to determine a minimum resistance in its conduction state, negligible in relation to the additional resistance $R_{A}$ that modifies the time constant of discharge (from $T_{e1}$ to $T_{e2}$).

Note that, from the viewpoint of Equation (4), $R_{L}$ represents $R_{ext1}$, and $R_{L}$ ‖ $R_{A}=50\;\Omega$ represents $R_{ext2}$.

For the first stage, we chose the discharging time interval $t_{s}-t_{p}≅10\;\mathrm{ms}$, approximately equal to the time constant of the circuit corresponding to the nominal capacitance and to $R_{ext1}$. Analogically, the time interval of the second stage was chosen $t_{f}-t_{s}≅5$ ms.

To establish the value of the sampling period, we performed, using the MATLAB environment, a Fourier analysis on the signal $y\left(t\right)$ corresponding to an entire discharge in only one stage. The result revealed a spectrum with significant components up to 25 kHz. For this reason, the processing of the signal $y\left(t\right)$ according to Shannon’s theorem requires a minimum sampling frequency of 50kHz and a sampling period of 20 μs, respectively. For this frequency, considering the range of the microcontroller conversion time 0.7 μs÷1.25 μs, it follows that the time left for digital processing is 20−1.25=18.75 μs. According to the microcontroller data sheet, the conversion error of the microcontroller is 3.4 LSB for 12 bits and 1.2 LSB for 8 bits [19]. The maximum sampling frequency is 420 kHz for 12-bit resolution. Due to these data, the maximum oversampling factor is (420/50) = 8 samples/processing period. Taking into consideration these aspects, we performed comparative experiments for 8-, 10- and 12-bit sampling resolutions in parallel with simple sampling technique and oversampling with averaging techniques [20]. Finally, we adopted 8× oversampling with a 12-bit resolution. That means an oversampling frequency of 400 kHz with 2.5 μs period.

**Figure 8:**
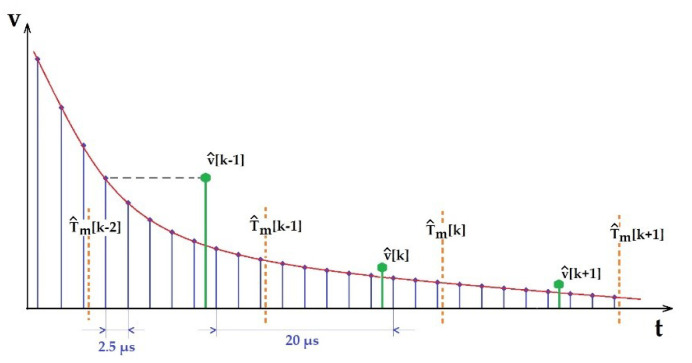
The timing of oversampling (**—**), sampling by averaging (**—**) and calculation of ${\hat{T}}_{m}$ (---) operations.

The timing of these operations is illustrated in Figure 8. The discharge voltage curve is depicted with the samples taken by the analog-to-digital converter. For every 8 samples averaged, a value $v\left[k\right]$ is used to calculate ${\hat{T}}_{m}\left[k\right]$.

As a result, we developed an application program running with an oversampling period of 2.5 μs and an equivalent sampling period h=20 μs. Mainly, a processing cycle contains the acquisition by oversampling, computing the average value $v\left[k\right]$ and computing ${\hat{T}}_{m}\left[k\right]$.

To perform the necessary sampling and processing operations, we used the ADC.h library included in the Teensyduino add-in [21]. Calling these parameters instantiates an object corresponding to the abovementioned values. The reading of the samples is performed in an interrupt service routine (ISR) triggered by an internal timer at 20 μs.

**Figure 9:**
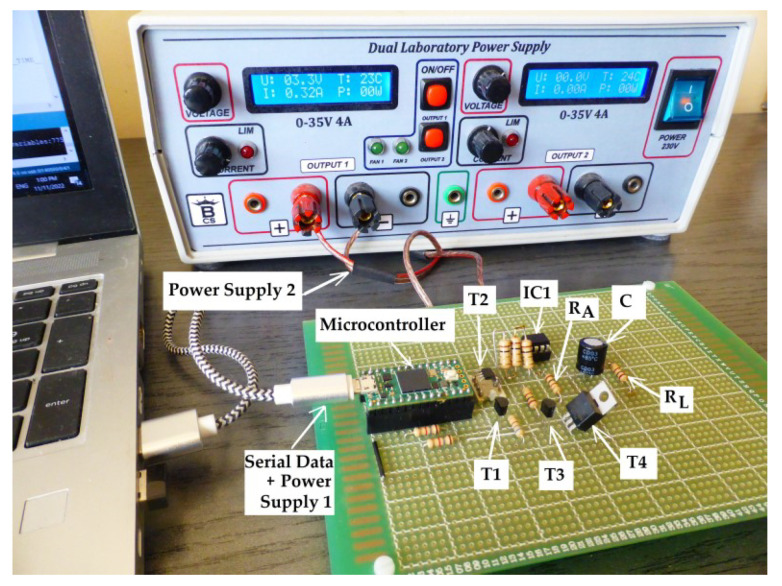
The test module for implementing *PO*.

The circuit in Figure 6 was implemented on a test module, as shown in Figure 9.

## 3. Results

To investigate the possibilities of experimentally determining the values of the parameters C and $R_{s}$ of a capacitor using the *PO*, experiments were carried out with three capacitors as follows:

- 1C— 100 μF/35 V, SR Passives, CE Series;
- 2C— 220 μF/35 V, Samxon, KM Series;
- 3C— 470 μF/35 V, Elite, EP Series.

The study was conducted on the basis of the following scenario: for each of the three capacitors, 5 series of 20 experiments were carried out with the schematic in Figure 6, consisting of repeating the scenario in Figure 3 at intervals of approximately 2 ÷ 3 min between two successive experiments. During all series of experiments, the temperature was approximately 22℃.

The main goal pursued in each individual series was the precision of the values obtained with the *PO* for ${\hat{T}}_{e1}$, ${\hat{T}}_{e2}$, $C_{e}$ and $R_{se}$. Table 1 refers to the results obtained. All values in this table are truncated. We have written in blue, black and, respectively, dark red the values obtained experimentally with the *PO* for the capacitors 1C, 2C and 3C. The columns written on a white background contain four average values ${\hat{T}}_{e1}$, ${\hat{T}}_{e2}$, $C_{e}$ and $R_{se}$, as well as four sample standard deviations related to the average values, expressed as a percentage according to Equation (6):

(6) $$\sigma_{\%}\left[x\right]=\frac{\sigma \left[x\right]}{\bar{\sigma }}\cdot 100,\sigma \left[x\right]=\sqrt{\frac{\sum_{i=1}^{n}{\left(x_{i}-\bar{x}\right)}^{2}}{n-1}},\;\;\;\;\;x\in \left\{{\hat{T}}_{e1},{\hat{T}}_{e2},C_{e},R_{se}\right\}.$$

The last four columns, written on a light-blue background, refer to the last experiment in each series of 20 experiments. The values ${\hat{T}}_{e1,20}$, ${\hat{T}}_{e2,20}$ were provided experimentally by the *PO*, and the values ${\hat{T}}_{e1,20}^{\prime }$, ${\hat{T}}_{e2,20}^{\prime }$ were calculated using off-line processing of the measured voltage $v\left[k\right]$. The off-line processing consisted of the regression generation for exponentials of shape $a_{1}\cdot e^{\frac{-t}{T_{e1}^{\prime }}}+b_{1}$ and $a_{2}\cdot e^{\frac{-t}{T_{e2}^{\prime }}}+b_{2},$. respectively, of values to approximate, on a least-squares basis, the experimental discharge curves in intervals III and V in Figure 3. We must note the very good correspondence, on the one hand, between the values of ${\hat{T}}_{e1,20}$ and ${\hat{T}}_{e1,20}^{\prime }$, and on the other hand, between the values of ${\hat{T}}_{e2,20}$ and ${\hat{T}}_{e2,20}^{\prime }$.

**Figure 10:**
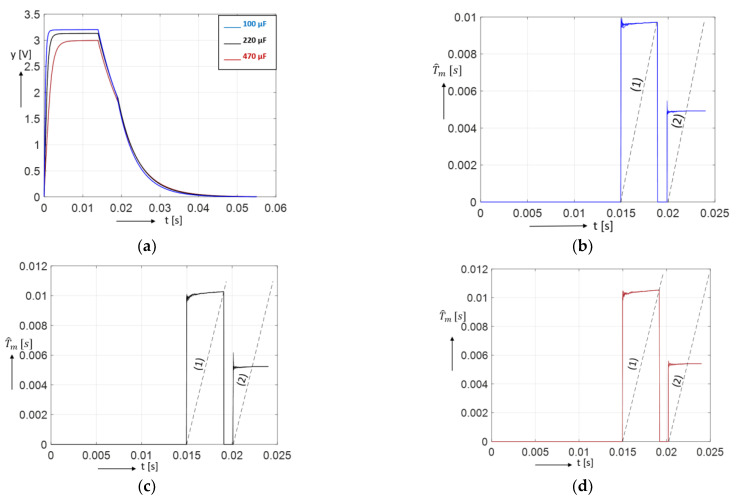
Dynamic characteristics used in processing the 20th experiment of each 5 series of experiments for capacitors 1C, 2C and 3C (the characteristics for every capacitor are practically overlapping). (**a**) The charging/discharging characteristics ${\left\{v\left[k\right]\right\}}_{k\geq 0,k\in N}$; (**b**–**d**) the characteristics ${\left\{{\hat{T}}_{m}\left[k\right]\right\}}_{k\geq k_{o}}$ used for calculation of ${\hat{T}}_{e1,20}$ and ${\hat{T}}_{e2,20}$ for 1C, 2C and 3C, respectively.

The charging/discharging characteristics ${\left\{v\left[k\right]\right\}}_{k\geq 0,k\in N}$ used for the last four columns of Table 1 are represented in Figure 10a. Figure 10b–d show the characteristic ${\left\{{\hat{T}}_{m}\left[k\right]\right\}}_{k\geq k_{o}}$. In all figures, at the chosen representation scale, no distinction can be made between the five characteristics derived from the five sets.

**Figure 11:**
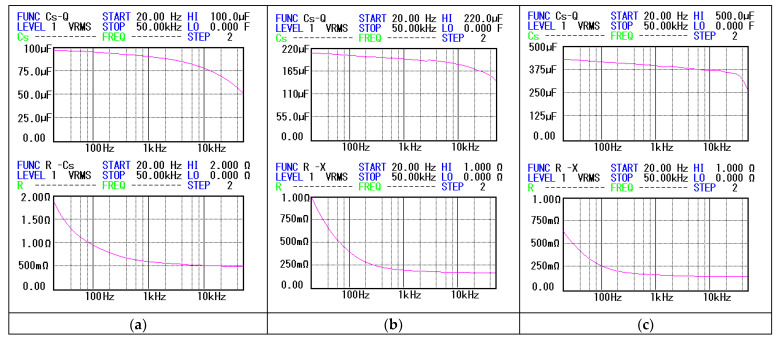
Frequency characteristics $C\left(f\right)$ and $R_{s}\left(f\right)$ of capacitors: (**a**) characteristics of capacitor 1C; (**b**) characteristics of capacitor 2C; (**c**) characteristics of capacitor 3C.

To qualitatively validate the accuracy of the results in Table 1, we used the frequency characteristics of the capacitors (Figure 11) determined by a BK Precision RLC-bridge ([14], Figure 7).

The main observations on the data in Table 1 and Figure 11 are shown in Table 2. In the table, we associated frequency intervals, sometimes restricted to a point, to the intervals in which ${\bar{C}}_{e}$ and ${\bar{R}}_{se}$ take the values in Table 1. For example, in the case of the 3C capacitor, the frequency interval $f\in \left(36.337.5\right)\mathrm{Hz}$ in Figure 11c (bottom) corresponds to the interval ${\bar{R}}_{se}\in \left(0.6820.706\right)\;\Omega$ in Table 1.

## 4. Discussion

1. The aim of our paper was to illustrate that the *PO* proposed in [11] can be implemented in real time. Implementation has several important attributes that are relevant for a wide range of processes. For example, from the point of view of power electronic applications, we highlight the following key advantages: a good speed of estimation, a reduced computational complexity and a good estimation accuracy [1]. Considering these aspects, we appreciate that the monitoring of capacitors using *PO*, being a quasi-online method, can be implemented on the same processor that already serves the process. We consider that our objective has been achieved, and we acknowledge this through the explanations below.
2. In Section 2.1, we summarized the procedure for determining the values of equivalent parameters of a capacitor using the *PO* proposed in [11], and we deepen the procedure regarding two aspects: (i) emphasizing the influence of the deviations of ${\hat{T}}_{e1}$ and ${\hat{T}}_{e2}$ in the result of calculating the equivalent values $C_{e}$ and $R_{se}$; (ii) extending the bisector’s method by rotating it, expressed by a coefficient α explained in Appendix A. Through (i), we highlight the very high sensitivity of the results obtained for $R_{se}$ in relation to deviations in ${\hat{T}}_{e1}$ and ${\hat{T}}_{e2}$, and we empirically argue for the use of a coefficient α=2.5.
3. The implementation of the *PO* is described in Section 2.2. The hardware support was an i.MX RT1062 microcontroller with Cortex^®^-M7 architecture. The necessary sampling and processing operations were performed using the ADC.h software component included in the Teensyduino add-in library. We must mention that in this paper, we present the main elements necessary for the reproduction of the application. Note that the cost of hardware support is low. In this context for the *PO*, we used a h=20 μs sampling period, anticipated in [11], and the acquisition process was performed with a 12-bit resolution and 8× oversampling technique.
4. The actual behavior of the implemented *PO* is illustrated in Section 3 for three capacitors with 100 μF, 220 μF and 470 μF nominal values. For each capacitor, we presented the results of 5 series of 20 experiments. First, the results show a low dispersion of the values of the equivalent time constants ${\hat{T}}_{e1}$ and ${\hat{T}}_{e2}$. Thus, the sample standard deviations related to the average values of ${\hat{T}}_{e1}$ and ${\hat{T}}_{e2}$ were in the ranges of 0.0482% ÷ 0.0921% and 0.0759% ÷ 0.1871%, respectively. This led, according to the discussion in Section 2.1.2, to limited intervals for sample standard deviations (0.1422% ÷ 0.2120 and 3.6594% ÷ 10.660%, respectively) of the average values of $C_{e}$ and $R_{se}$. Second, the calculated average values of $C_{e}$ and $R_{se}$ are found at frequencies below 180Hz in the measured frequency characteristics of the capacitors. Finally, we note that the off-line regression calculations for 15 two-stage discharge processes showed the same results as the results obtained in real time with *PO*.
5. The low dispersion highlights the potential of the *PO* for providing precise results, i.e., values of $C_{e}$ and $R_{se}$ with a low scattering. Simultaneously, considering $C_{e}$ and $R_{se}$ values in relation to their frequency characteristics suggests the potential of the method for obtaining accurate results. The scattering of results is due both to the fact that the discharge processes obey statistical laws and the fact that the errors appear in the sampling of the measured values of the voltage at the capacitor terminals. We consider that by using better hardware and software resources in applications, both precision and accuracy can be improved.
6. The experiments described in this paper were performed on independent capacitors. This approach may also be found in other research, for instance, the work in [22]. The use of *PO* in real applications involves ensuring the quasi-online estimation framework used in this paper [23]. On the one hand, this requires delimitating a very short time for charging/discharging the capacitor, and on the other hand, it is necessary to provide the resistances over which the capacitor is discharged in the two stages in the electrical circuit of the application and to accurately determine its values. The first requirement is met by processes that are not in a continuous operating regime, for example, in the motor driver converter during the stop of the motor driver, solar PV or in processes with intermittent operation. The second requirement is achievable using suitable switching circuits.
7. It should also be noted that the experiments reported in this article correspond to some discharges of capacitors during which the voltage spectrum on the capacitor is predominantly at relatively low frequencies. The frequency ranges in Table 2 corroborate this.

## 5. Conclusions

For reasons of safety in operation and maintenance, numerous systems require the monitoring of capacitors placed in key positions in their electrical diagrams. The parameter observer whose implementation is the subject of this paper can serve the mentioned requirement in real time. It requires ensuring the possibility of discharging the monitored capacitor over a well-known variable resistor in each of the two stages.

The implementation carried out on a common microcontroller illustrates that the method has good precision and fairly good accuracy in terms of determining the parameters of some electrolytic capacitors subjected to signals with not very high frequency spectra. The sampling time with which the voltage signal was acquired from the capacitor terminals and with which the parameter observer worked was 20 μs, and the estimation of the capacitor parameters was fulfilled within the time allocated to the discharge of the capacitors. The *PO*-estimated values of C and ESR are consistent with the frequency characteristics measured with an LCR bridge.

## Figures and Tables

**Table 1:** Experimental results for the capacitors 1C ($R_{L}=R_{a}=98.9\;\Omega$), 2C ($R_{L}=R_{a}=49.5\;\Omega$), 3C ($R_{L}=44.8\;\Omega ,R_{a}=24.8\;\Omega$). The following settings were used: $t_{p}=14\;\mathrm{ms}$, ($k_{p}=700$), $t_{o}=1\;\mathrm{ms}$, ($k_{o}=50$), $t_{f}=40\;\mathrm{ms}$, ($k_{f}=2000$), α=2.5.

**No. of Series of** **Experiments**	${\bar{\hat{T}}}_{e1}$ [ms]	${\bar{\hat{T}}}_{e2}$ [μs]	${\bar{C}}_{e}$ $\left[\mu F\right]$	${\bar{R}}_{se}$ $\left[\Omega \right]$	$\sigma_{{\hat{T}}_{e1}\%}$ [%]	$\sigma_{{\hat{T}}_{e2}\%}$ [%]	$\sigma_{C_{e}\%}$ [%]	$\sigma_{R_{se}\%}$ [%]	${\hat{T}}_{e1,20}$ [μs]	${\hat{T}}_{e2,20}$ [μs]	${\hat{T}}_{e1,20}^{\prime }$ [μs]	${\hat{T}}_{e2,20}^{\prime }$ [μs]
1	9.7106	4.9235	96.806	1.41	0.0654	0.1323	0.1869	10.660	9.7157	4.9235	9.6736	4.9581
	10.274	5.2278	203.89	0.890	0.0582	0.0964	0.1675	7.0825	10.266	5.2333	10.224	5.2890
	10.536	5.4137	413.08	0.706	0.05655	0.1138	0.1671	4.8510	10.533	5.4087	10.474	5.2890
2	9.7167	4.9213	96.974	1.299	0.0654	0.1323	0.1869	9.0055	9.7149	4.9181	9.6760	4.9646
	10.269	5.2251	203.81	0.887	0.0482	0.1575	0.1905	9.7135	10.266	5.2158	10.217	5.2798
	10.551	5.4171	413.96	0.686	0.0664	0.1028	0.1422	3.9733	10.548	5.4203	10.482	5.4691
3	9.7231	4.9224	97.082	1.254	0.0598	0.0759	0.1425	7.8331	9.7234	4.9247	9.6845	4.9684
	10.270	5.2275	203.76	0.905	0.0921	0.1082	0.2120	7.9177	10.272	5.2256	10.222	5.2887
	10.536	5.4123	413.23	0.698	0.0519	0.1871	0.2078	7.1817	10.538	5.4125	10.467	5.4687
4	9.7120	4.9200	96.907	1.321	0.0798	0.0970	0.2211	11.528	9.7082	4.9203	9.6772	4.9628
	10.270	5.2271	203.75	0.904	0.0575	0.1085	0.1739	7.5261	10.271	5.2279	10.217	5.2896
	10.537	5.4097	413.52	0.682	0.0557	0.1027	0.1765	5.0745	10.541	5.4075	10.474	5.4846
5	9.7115	4918.2	96.933	1.288	0.0662	0.0999	0.1632	9.2708	9.7110	4.9214	9.6732	4.9532
	10.274	5.2289	203.84	0.901	0.0705	0.1110	0.1568	6.5866	10.268	5.2272	10.222	5.2809
	10.534	5.4096	413.31	0.689	0.0705	0.0766	0.1518	3.6594	10.532	5.4032	10.472	5.4621

**Table 2:** The association of frequency intervals in Figure 11 with intervals in which ${\bar{C}}_{e}$ și ${\bar{R}}_{se}$ in Table 1 take values.

1C	2C	3C
(96.907 ÷ 97.082) μF/(30 ÷ 37) Hz	(203.75 ÷ 203.89) μF/106.2 Hz	(413.08 ÷ 413.96) μF/178.8 Hz
(1.254 ÷ 1.41) Ω/(34 ÷ 45) Hz	(0.887 ÷ 0.905) Ω/24 Hz	(0.682 ÷ 0.706) Ω/(36.3 ÷ 37.5) Hz

**Figure A1:**
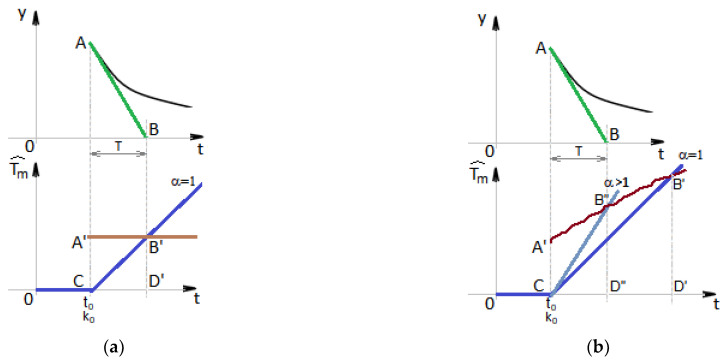
Regarding the application of the bisector method: (**a**) the ideal case; (**b**) the real case.

**Figure A2:**
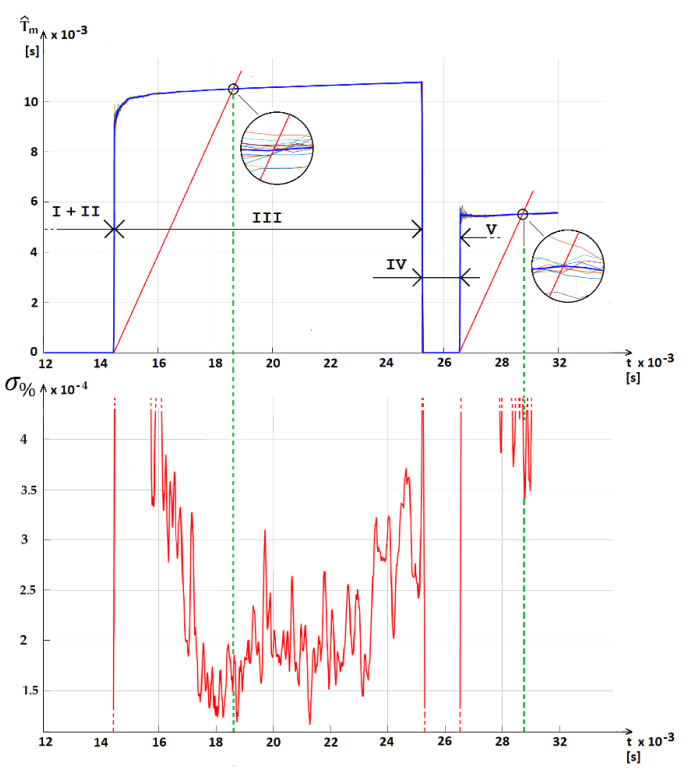
Regarding the dispersion of experimental results’ processing.

**Figure A3:**
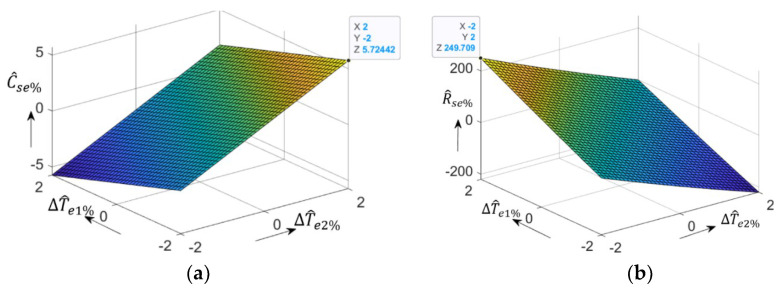
The percentage deviations of $C_{e}$ and $R_{se}$ from the ideal ones for a capacitor with ${\hat{C}}_{e0}=100\;\mu F,R_{se0}=1.8\;\Omega$, ${\hat{T}}_{e10}=0.01018\;s,\;{\hat{T}}_{e20}=0.00518\;s,\;R_{ext1}=100\;\Omega ,R_{ext2}=50\;\Omega$; (**a**) deviation ${\hat{C}}_{e\%}$; (**b**) deviation ${\hat{R}}_{se\%}$.

**Figure A4:**
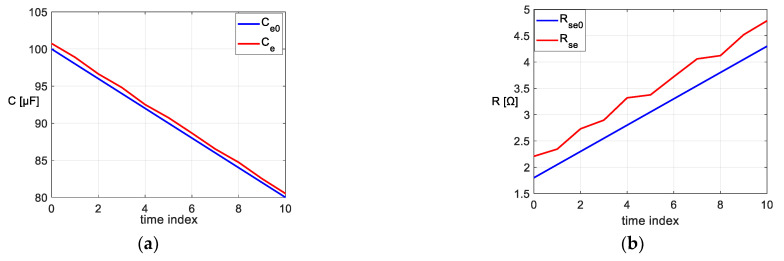
Concerning the conservation of the monotony of the variations of $C_{e}$ and $R_{se}$ during the monitoring processes affected by the calculation errors of ${\hat{T}}_{e1}$ and ${\hat{T}}_{e2}$ in line with Table A1: (**a**) variations ${\hat{C}}_{e0}\left(t\right)$ and ${\hat{C}}_{e}\left(t\right)$; (**b**) variations ${\hat{R}}_{se0}\left(t\right)$ and ${\hat{R}}_{se}\left(t\right)$.

## Data Availability

Not applicable.

## Abbreviations

ESR	equivalent serial resistance
*PO*	parameter observer

## Appendix A

To discuss the application of the bisector method, we consider Figure A1. The figure shows two cases of processing a discharge in one step using the structure in Figure 1b. Cases are denoted by (a) and (b). In both cases, the signal $y\left(t\right)$ is the same, namely, a purely exponential signal. This does not happen with a real signal, so y(t) can be interpreted in case (b) as an exponential signal equivalent to the real signal. The signal ${\left\{{\hat{T}}_{m}\left[k\right]\right\}}_{k\geq k_{o}}$ is obtained by averaging the *PO* output according to Equation (5).

The two cases differ by the degree of signal alteration ${\left\{{\hat{T}}_{m}\left[k\right]\right\}}_{k\geq k_{o}}.$ In case (a), the processing is not altered, and as a result, ${\left\{{\hat{T}}_{m}\left[k\right]\right\}}_{k\geq k_{o}}=const.$ In case (b), the processing is altered, and according to the experiments carried out, the characteristic ${\left\{{\hat{T}}_{m}\left[k\right]\right\}}_{k\geq k_{o}}$ presents an increasing tendency during the discharging process. This is due to the deviation in the real signal from the exponential and the disturbances that appear in the measuring and the processing chain of $y\left(t\right)$, starting with the acquisition process and ending with the application of Equation (5).

The bisector method serves to estimate $T_{e}$ in real time from the calculated values ${\left\{{\hat{T}}_{m}\left[k\right]\right\}}_{k\geq k_{o}}$. The name of the method is given by the case (a), and the method boils down to calculating the estimate ${\hat{T}}_{e}$.

(A1) $${\hat{T}}_{e}=k_{e}\cdot h,$$

where $k_{e}$ is the solution of the equation

(A2) $${\hat{T}}_{m}\left[k_{o}+k_{e}\right]≅\alpha \cdot k_{e}\cdot h$$

for α=1. The left member represents the length of the segment $B^{\prime }D^{\prime }$, and the right member represents the length of the segment $CD^{\prime }$, so graphically, the point $B^{\prime }$ results at the intersection of the bisector of the angle $\hat{A^{\prime }CD^{\prime }}$ with the characteristic ${\left\{{\hat{T}}_{m}\left[k\right]\right\}}_{k\geq k_{o}}.$ The bisector method is applied by checking at each calculation step k the condition $\left|B^{\prime }D^{\prime }-CD^{\prime }\right|\leq h$ or

(A3) $$\left|{\hat{T}}_{m}\left[k_{o}+k\right]-\alpha \cdot k\cdot h\right|\leq h,\;\;\;\;\;k>k_{o}.$$

Since h represents the discretization step of the *PO*, the inequality (A3) has a unique solution $k_{e}$, so the last value used in checking the condition (A2), ${\hat{T}}_{m}\left(k_{e}\cdot h\right),$ is considered the value of ${\hat{T}}_{e}$.

In case (b), the application of relation (A1) for α = 1 leads to an erroneous result, since the point $B^{\prime }$ corresponds to a segment $CD^{\prime }$ of length greater than T, and so a ${\hat{T}}_{e}$ of a value greater than the expected one results. A better approximation of T can be obtained either by translating the bisector $CB^{\prime }$ to the left, as in Figure 2f, or by rotating it anticlockwise to the $CB^{\prime \prime }$ position, as in Figure A1b. The rotation means adopting a coefficient α>1 in Equation (A1).

The value of α depends both on the signal $y\left(t\right)$ and on the hardware and software on which the *PO* is implemented. As a result, the adoption of the value α can only be achieved empirically, per the type of application, without having the certainty that ${\hat{T}}_{m}\lceil k_{o}+k_{e}\rceil =T.$

In the experiments, we adopted α=2.5. The choice was made based on the observation of the dispersion of the characteristics $\left\{{\hat{T}}_{m}\left[k\right]\right\}$ obtained on the set of whole discharges in two stages of the capacitors. This idea is exemplified in Figure A2, which refers to a sample of 15 two-stage discharges of capacitor 3C. In the upper part of the figure, the characteristics $\left\{{\hat{T}}_{m}\left[k\right]\right\}$ are illustrated. The characteristic obtained for the average value is drawn in black. Notations I−V have the same meaning as for Figure 3. A dispersion of characteristics is suggested in medallions. The lower part of Figure A2 represents the sample standard deviations related to the average values $\sigma_{\%}\left[{\hat{T}}_{m}\left[k\right]\right]$. The domain in which the dispersion has the lowest values is centered, both in zone III and in zone V, around the positions of the line $CB^{\prime \prime }$ corresponding to αϵ⌈2,3⌉.

## Appendix B

Equation (4) presents sensitivities of different orders of magnitude in relation to the values calculated for ${\hat{T}}_{e1}$ and ${\hat{T}}_{e2}$. To discuss this aspect, we assume that ${\hat{T}}_{e10}$ and ${\hat{T}}_{e20}$ are the values of the two equivalent time constants corresponding to ideal conditions for measurement and subsequent processing of the voltage values at the capacitor terminals. In the following, we call „ideal values” all values that have the subscript „0”. Let $C_{e0}$ and $R_{se0}$ be the ideal values obtained by substituting ${\hat{T}}_{e10}$ and ${\hat{T}}_{e20}$ in (4), respectively, and $\Delta {\hat{T}}_{e1}$ and $\Delta {\hat{T}}_{e2}$ are the deviations of the measured values from the ideal values, i.e., ${\hat{T}}_{e1}={\hat{T}}_{e10}+\Delta {\hat{T}}_{e1}$ and ${\hat{T}}_{e2}={\hat{T}}_{e20}+\Delta {\hat{T}}_{e2}$. We introduce the quantities (A4) with the meaning of percentage deviations of ${\hat{T}}_{e1}$, ${\hat{T}}_{e2}$, $C_{e}$ and $R_{se}$ from the ideal ones and the ratios in (A5):

(A4) $$\Delta {\hat{T}}_{e1\%}=\frac{\Delta {\hat{T}}_{e1}}{{\hat{T}}_{e10}}\cdot 100,\;\Delta {\hat{T}}_{e2\%}=\frac{\Delta {\hat{T}}_{e2}}{{\hat{T}}_{e20}}\cdot 100,\;{\hat{C}}_{e\%}=\frac{{\hat{C}}_{e}-C_{e0}}{C_{e0}}\cdot 100,\;{\hat{R}}_{se\%}=\frac{{\hat{R}}_{se}-R_{se0}}{R_{se0}}\cdot 100.$$

(A5) $$k_{T}=\frac{{\hat{T}}_{e10}}{{\hat{T}}_{e20}},\;\;\;k_{R}=\frac{R_{ext1}}{R_{ext2}}.$$

With these notations, we obtain the equalities:

(A6) $${\hat{C}}_{e\%}=\frac{k_{T}\cdot \Delta {\hat{T}}_{e1\%}-\Delta {\hat{T}}_{e2\%}}{k_{T}-1},\;\;{\hat{R}}_{se\%}=\frac{\left(k_{R}-1)\cdot (\Delta {\hat{T}}_{e2\%}-\Delta {\hat{T}}_{e1\%}\right)}{(k_{R}-k_{T})\cdot \left[(1+0.01\cdot \Delta {\hat{T}}_{e1\%})-\frac{1+0.01\cdot \Delta {\hat{T}}_{e2\%}}{k_{T}}\right]}$$

Considering that:

(A7) $$k_{R}-k_{T}≅\frac{R_{se0}}{R_{ext2}},$$

the second Equation (A6) becomes:

(A8) $${\hat{R}}_{se\%}≅\frac{R_{ext2}}{R_{se0}}\cdot \frac{\left(k_{R}-1)\cdot (\Delta {\hat{T}}_{e2\%}-\Delta {\hat{T}}_{e1\%}\right)}{(1+0.01\cdot \Delta {\hat{T}}_{e1\%})-\frac{1+0.01\cdot \Delta {\hat{T}}_{e2\%}}{k_{T}}}.$$

As the value of the first fraction (A8) is very large, and the second one is comparable to the value of the fraction in the expression of ${\hat{C}}_{e\%},$ it follows that ${\hat{R}}_{se\%}\gg {\hat{C}}_{e\%}$. This is evidenced by the example in Figure A3. Let us observe that for variations $\Delta {\hat{T}}_{e1\%}\in \left[-2\%,2\%\right]$ and $\Delta {\hat{T}}_{e2\%}\in \left[-2\%,2\%\right],$ maximum deviations of +6.72442% occur for $C_{e},$ and +249.709% for $R_{se}$.

Table A1 shows the hypothetical monitoring situation of an ageing process of the capacitor in Figure A3 over 11 equidistant moments, during which the value $C_{e0}$ falls from 100 μF to 8 0 μF, and the value $R_{se0}$ rises from 1.8 Ω to 4.3 Ω. The errors $\Delta {\hat{T}}_{e1\%}$ and $\Delta {\hat{T}}_{e2\%}$ show moderate variations during the monitoring moments. Figure A4 represents the results graphically. We observe that the monotony of the variation of the two parameters is maintained even in the presence of errors $\Delta {\hat{T}}_{e1\%}$ and $\Delta {\hat{T}}_{e2\%}$.

**Table A1:** Example concerning the conservation of the monotony of the variation of $C_{e}$ and $R_{se}$.

t	0	1	2	3	4	5	6	7	8	9	10
$C_{e0}\left[\mu F\right]$	100	98	96	94	92	90	88	86	84	82	80
$R_{se0}\left[\Omega \right]$	1.8	2.05	2.3	2.55	2.8	3.05	3.3	3.55	3.8	4.05	4.3
$\Delta {\hat{T}}_{e1\%}$	1.15	1.2	1.09	1.21	1.06	1.14	1.165	1.098	1.17	1.08	1.12
$\Delta {\hat{T}}_{e2\%}$	1.54	1.48	1.495	1.533	1.541	1.44	1.55	1.56	1.46	1.5	1.55
$C_{e}\left[\mu F\right]$	100.74	98.89	96.64	48.82	92.51	90.74	88.66	86.52	84.72	82.51	80.52
$R_{se}\left[\Omega \right]$	2.208	2.344	2.73	2.895	3.319	3.375	3.72	4.059	4.121	4.519	4.784
